# Comprehensive Genomic Review of TCGA Head and Neck Squamous Cell Carcinomas (HNSCC)

**DOI:** 10.3390/jcm8111896

**Published:** 2019-11-07

**Authors:** Mario Pérez Sayáns, Cintia Micaela Chamorro Petronacci, Alejandro Ismael Lorenzo Pouso, Elena Padín Iruegas, Andrés Blanco Carrión, José Manuel Suárez Peñaranda, Abel García García

**Affiliations:** 1 Health Research Institute Foundation of Santiago (FIDIS); Oral Medicine, Oral Surgery and Implantology Unit, Faculty of Medicine and Dentistry, University of Santiago de Compostela, C.P. 15782 Santiago de Compostela, Spain; cintiamica.chamo@yahoo.es (C.M.C.P.); alexlopo@hotmail.com (A.I.L.P.); andres.blanco@usc.es (A.B.C.); abel.garcia@usc.es (A.G.G.); 2 Area of Human Anatomy and Embryology, Faculty of Physiotherapy, Department of Functional Biology and Health Sciences, University of Vigo, 36310 Vigo, Pontevedra, Spain; mepadin@ghotmail.com; 3 Pathological Anatomy Service, University Hospital Complex of Santiago (CHUS), C.P. 15782 Santiago de Compostela, Spain; jm.suarez.penaranda@gmail.com

**Keywords:** head and neck neoplasms, DNA copy number variation, genomics, oncogenes, differentially expressed genes

## Abstract

The aim of this present study was to comprehensively describe somatic DNA alterations and transcriptional alterations in the last extension of the HNSCC subsets in TCGA, encompassing a total of 528 tumours. In order to achieve this goal, transcriptional analysis, functional enrichment assays, survival analysis, somatic copy number alteration analysis and somatic alteration analysis were carried out. A total of 3491 deregulated genes were found in HNSCC patients, and the functional analysis carried out determined that tissue development and cell differentiation were the most relevant signalling pathways in upregulated and downregulated genes, respectively. Somatic copy number alteration analysis showed a “top five” altered HNSCC genes: CDKN2A (deleted in 32.03% of patients), CDKN2B (deleted in 28.34% of patients), PPFIA1 (amplified in 26.02% of patients), FADD (amplified in 25.63% of patients) and *ANO1* (amplified in 25.44% of patients). Somatic mutations analysis revealed *TP53* mutation in 72% of the tumour samples followed by *TTN* (39%), *FAT1* (23%) and *MUC16* (19%). Another interesting result is the mutual exclusivity pattern that was discovered between the *TP53* and *PIK3CA* mutations, and the co-occurrence of CDKN2A with the *TP53* and *FAT1* alterations. On analysis to relate differential expression genes and somatic copy number alterations, some genes were overexpressed and amplified, for example, FOXL2, but other deleted genes also showed overexpression, such as CDKN2A. Survival analysis revealed that overexpression of some oncogenes, such as EGFR, CDK6 or CDK4 were associated with poorer prognosis tumours. These new findings help us to develop new therapies and programs for the prevention of HNSCC.

## 1. Introduction

Head and neck squamous cell carcinomas (HNSCCs) encompass a heterogeneous group of malignancies that begin in the upper aerodigestive tract mucosae. HNSCCs include cancers of the oral cavity, larynx, and oropharynx [1,2]. According to the GLOBOCAN project, HNSCCs are the sixth most common cancers worldwide, with an annual estimation of 355,000 attributable deaths and 633,000 incident cases [3]. In this vein, the overall five-year survival rate for HNSCCs has not decreased in recent decades and it is still about 60% [4]. Treatment of HNSCCs relies on different combinations of surgery, radiotherapy, and chemotherapy. The rationale behind therapy decision-making has been classically based on TNM staging and the site involved; nonetheless, in recent years, the usefulness of the presence of high-risk human papillomavirus (HR-HPV) infection has been proven [5]. Tobacco use and alcohol intake are the best-characterised modifiable risk factors of HNSCCs, although its magnitude factors may change significantly within the variation of each subgroup [6]. 

Until 2010, very few efforts had been made in order to unravel the molecular biology of HNSCCs [7]. Recent initiatives have addressed this issue. Among these, the Cancer Genome Atlas (TCGA) consortium has provided the largest publicly available HNSCC dataset, which is considered to be a cornerstone for future research [8]. The impact of differentially expressed genes (DEGs) on the aggressiveness and evolution of tumours has revealed new roles which contribute to carcinogenesis. Today’s primary challenge is to differentiate between somatic mutations with oncogenic potential and all of those which crop up later in a tumour’s lifetime without contributing to the tumour’s development. In this sense, the concept of positive selection has opened a new avenue for the discovery of novel tumorigenic roles in cancer. Mutations that improve the fitness of the tumour cells would be under positive selection pressure, meaning, therefore, that they would recur at elevated rates across patients. This principle is widely used to identify these signals and differentiate them from alterations with real oncogenic power [2,9]. Since somatic genomic alterations are still considered to be very important oncogenic events, changes in tumour transcriptomes have now outdated the former classification of oncogenes which was based exclusively on their mutational profile [10]. The differences in mRNA expression is a new and important point for (1) discovering new signatures related to patients’ responses to drugs or treatments, (2) finding out potential oncogenic events and (3) providing further understanding of tumour cell signalling and the adaptability of tumour cells to changing environments. Nevertheless, tumour staging for HNSCC based on transcriptional signatures is not commonly used in treatment decision-making, although it can be very informative from a biological perspective [11,12]. In the past, sequencing technology was limited by the huge amount of generated data that required high levels of computational expertise, nowadays, however, bioinformatic advances have provided the possibility for this information to be integrated, leaving a huge window of opportunity for modern oncology [13]. 

The aim of the present study was to comprehensively describe somatic DNA alterations and transcriptional alterations in the last extension of the HNSCC subsets in TCGA, encompassing a total of 528 tumours.

## 2. Material and Methods

### 2.1. Samples and Clinical Data

Data from TCGA-HNSCC patients was collected in a database that had been specifically designed for this purpose, with repeated verification. The clinical variables used were age, sex, tobacco consumption, alcohol consumption, tumour stage and HPV (human papilloma virus) infection. The HPV infection status was determined according to the TCGA network group 2015 classification, using an empiric definition of >1000 mapped RNA sequencing (RNA-Seq) reads, primarily aligning to viral genes E6 and E7.

### 2.2. Transcriptional Analysis 

RNA-seq counts were downloaded from the Genomic Data Commons (GDC) data portal. The normalisation and differential expression analyses were then calculated following the instructions detailed in the TCGAbiolinks R package [14]. This package uses a first within-lane normalization to adjust for GC-content effect on read counts, and a second between-lane normalization to adjust for distributional differences. Differentially expressed genes were considered as those which had a (log2(FC) > 1) and false discovery range (FDR) *q*-value < 0.01 when comparing the mRNA level expression between the TCGA-HNSCC tumour and the normal samples. “exactTest” methods were used to calculate the *p*-value FDR correction that computes a gene-wise exact test for differences in the means between two groups of negative-binomially distributed counts. 

Survival analysis was performed by computing all the genes of the human transcriptome to see which of them can have an impact on HNSCC patients and in what way (if greater or lesser expression) it correlates with worse survival. After that, we have filtered data to show only those that have been linked to cancer and, therefore, fall under the classification of COSMIC cancer gene census (CGC).

### 2.3. Functional Enrichment Assays

Differentially expressed genes were divided into up- or down-regulated groups. Both lists were then used for functional annotation into biological process categories. For this purpose, we used the Metascape web app express analysis mode. This app allows the user to use expression information to determine how GO categories are differentially affected by higher or lower expressed genes in tumour samples.

### 2.4. Somatic Copy Number Alteration (SCNA) Analysis

The performance of the GISTIC algorithm will not be revised in this paper. In summary, we downloaded GISTIC results from the Broad Institute’s latest data Firehose run. Significant peak alterations were identified as those with a *q*-value < 0.25, as described in GISTIC documentation. Using threshold data, we were able to classify each patient for each gene into deeply amplified (+2), diploid (+1, 0 and −1) or deeply deleted (−2). The genes with alterations which affected more than 10% of the TCGA-HNSCC patients were considered as positively selected oncodriver alterations.

### 2.5. Somatic Alteration Analysis

Both the results from the MuTect2 and the VarScan variant callers were downloaded from the GDC data portal using the TCGAbiolinks R package (R version 3.6.1, Vienna, Austria). Mutation annotation format (MAF) files were concatenated and filtered from the duplicated and silent mutations using the Maftools R package. Likewise, all of the mutation data calculations were performed with this last package.

## 3. Results

### 3.1. Samples and Clinical Data

The TCGA-HNSCC cohort included 528 tumour samples resected from different locations across the patients’ heads and necks. This cohort consisted primarily of tumours from the oral cavity (72.40%), larynx (21.96%) and oropharynx (5.64%). In fact, most of the samples came from the tongue, tonsil and larynx (91.24% of the total 528 samples).

With regards to the patients’ data, there was a larger male than female representation (73.11% versus 26.89%, respectively) and samples from 50 to 70-year-old subjects (62.12% of the whole cohort) were the most abundant. In terms of habits, 76.70% of patients were smokers or former smokers, and 66.66% had a history of alcohol consumption. Only tumours in 38 patients (7.19%) could not be associated with either of these two unsafe habits.

This cohort included a large number of stage IVA (257 out of 528, 48.67%), stage III (82 out of 528, 15.53%) and stage II (74 out of 528, 14.02%) tumours, and only one from stage IVC (0.18%). With regards to the primary diagnostic, 85.04% of the tumours were diagnosed as squamous cell carcinomas (SCCs), while 10.80% were classified as keratinizing SCCs (SCCs-K). Other types were: SCCs from large cell and not keratinizing (11 out of 528, 2.08%), SCCs basaloids (1.89%) and SCCs from spindle cells (1 out of 528, 0.18%—the only one classified as stage IVC).

With regards to the HPV infection status, we were able to conclude that the current TCGA-HNSC cohort was formed by 36 HPV (+), 243 HPV (−) and 249 HPV “unknown” status samples. Regarding the resection site, the highest proportion of HPV (+) were found in the tonsil (78.94%), whereas the least common areas were the cheek mucosa, lip, mouth and oropharynx, with no infected samples. The second and third positions were BOT (base of tongue) (54.54%) and hypopharynx (50%), respectively.

### 3.2. Transcriptional Alterations and Functional Enrichment Assays

**Figure 1:**
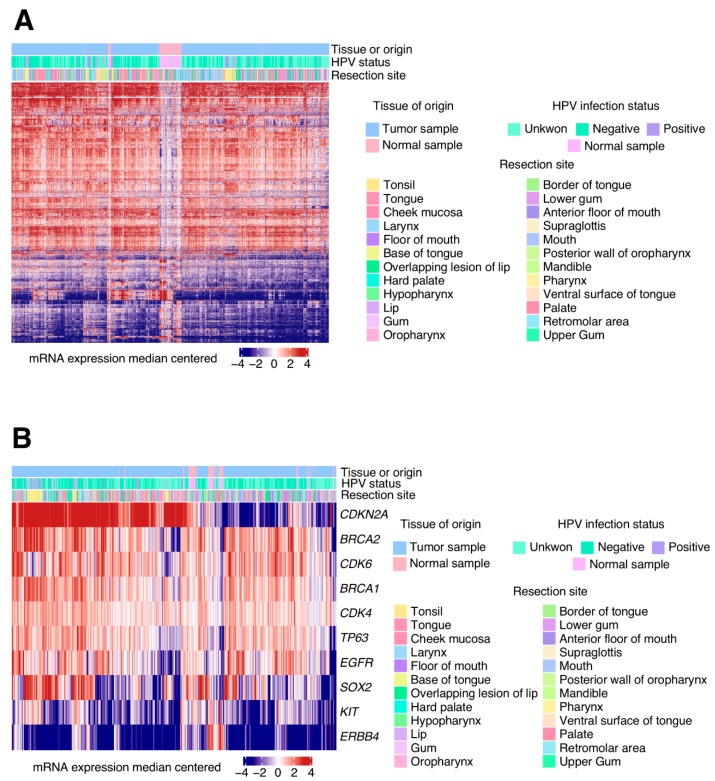
mRNA expression for each gene was computed into the Cancer Genome Atlas (TCGA)-head and neck squamous cell carcinomas (HNSCCs) tumour and normal samples. (**A**) Differentially expressed genes z-score heat map comparing the mRNA levels for tumours and normal samples. Hierarchical clustering shows the associations between the samples which share similar alteration patterns. This figure also shows information which makes an association between the human papillomavirus (HPV) infection status and the resection sites. (**B**) Heatmap of *z*-score for relevant genes involved in HNSCC pathogenesis that are differentially expressed between the normal and tumour samples. Clinical information has also been added to better differentiate altered patterns according to the HPV infection status or resection site.

In our study, we identified 3491 DEGs in HNSCC tumour samples when compared to healthy oral tissue. In Figure 1A, we can see the origin of tumour samples used. HPV infection status and Hierarchical clustering shows the associations between the samples which share similar alteration patterns, for example, we can observe how normal samples are characterized by visual different gene expression (see white middle band in the figure that represents no different fold-change expression). 

Further analysis indicated the presence of differentially expressed cancer-related genes. In total, 148 oncogenes and tumour suppressors from the COSMIC cancer genes census list were deregulated in these tumours. We highlighted different expression concretely in 10 genes that are commonly known to be deregulated in HNSCC (Figure 1B). For example, samples from base of tongue, that are concentrated in the left site of the figure, clearly show a high expression of CDKN2A compared to normal samples.

**Figure 2:**
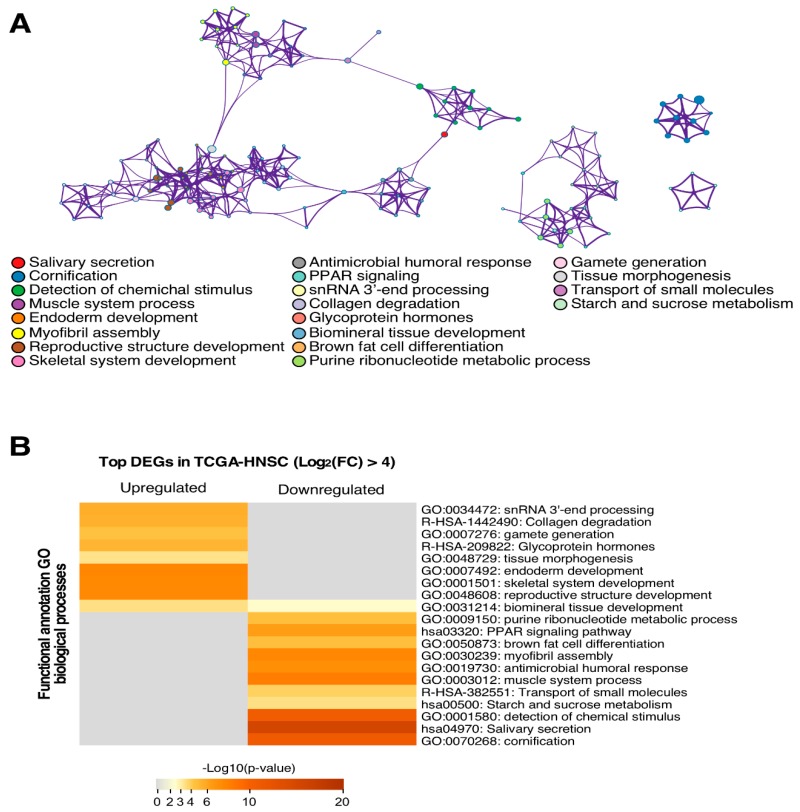
The functional annotation analysis shows the association of the genes with several biological processes. (**A**) Top differentially expressed genes both up- or down-regulated were annotated functionally in GO terms derived from the biological process. Each GO term is represented as a node and links show the gene sharing level between two nodes. Each colour represents one GO term as indicated in the legend. (**B**) GO terms are segregated depending on when they are more likely to be affected by up- or down-regulated genes. The heat map indicates the significant level of each term annotation depending on the number of genes contained from the input list.

The functional annotation of the top transcriptionally altered genes (log (FC) > 4) demonstrated their integration in tumour-relevant signalling pathways. In particular, we found upregulated genes involved in tissue development and the repression of those implicated in cell differentiation (Figure 2). There were also other genetic circuits enriched in these DEGs involved in cellular matrix reorganization or metabolic rewiring, both of which are important activities for tumour cell fitness.

We also performed gene expression profile attending on tissue origin. We grouped tumour samples according to their localization, based on risk factors and molecular mechanisms implicated, in oral cavity (tongue, cheek mucosa, floor of mouth, base of tongue, hard palate, gum, lower gum, mouth, anterior floor of mouth, mandible, border of tongue, retromolar area, upper gum, palate, ventral surface of tongue), lip (lip, overlapping lesion of lip), oropharynx tonsil, oropharynx, supraglotis, posterior wall of orophayrnx, pharynx and hypopharynx (laringe, hypharynx). A total of 286 samples belonged to the oral cavity group, 55 to oropharynx, 137 to hypopharynx, and 86 to lip. If we select, only those genes that are differentially expressed according to their localization, we observed that in the oral cavity, we obtained 133 genes, in oropharynx 1209 genes, in hypopharynx 224, and in lip 86. To visualize the different profiles obtained in each group, the 20 most relevant DEGs, attending to *p*-value, from each group are summarized in Table 1, Table 2, Table 3 and Table 4.

Survival analysis revealed 25 genes, that are DEGSs in HNSCC and that can be associated significantly with poorer prognosis. The majority of them were identified as oncogenes, and only 4 of them could be identified as tumour suppressor gene (Table 5).

We have also compared expression levels in HPV (+) samples compared with HPV (−) samples. Although we have obtained more than 2000 DEGs in this analysis, we resumed more significantly differently expressed genes in Table 6.

### 3.3. Somatic Copy Number Alterations

**Figure 3:**
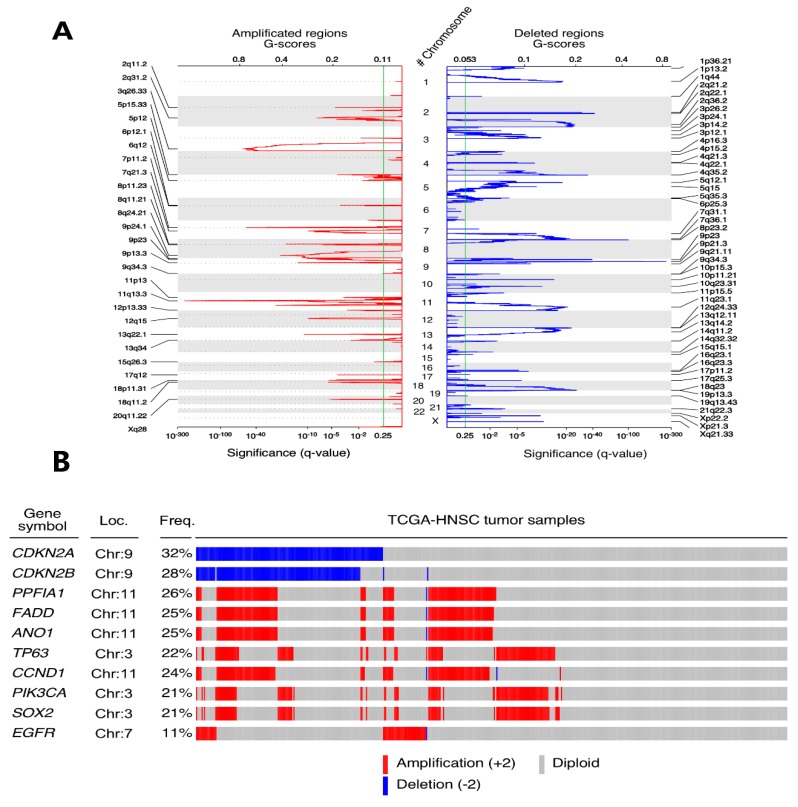
The GISTIC algorithm results reveal alterations of different genes at copy number level. (**A**) The chromosomal location of these alterations is represented as peaks in which the height over the *χ*-axis represents the significant *q*-value calculated and the width on the *Υ*-axis is associated with the genomic extension affected by this change. (**B**) The Top COSMIC cancer gene census list altered in the TCGA-HNSCC cohort are represented in the oncoplot. Blue is associated with a deep deletion in a patient of a particular gene whereas red represents a deep amplification. The chromosome location is provided as well as the percentage (%) of patients affected with each gene’s somatic copy number alteration (SCNA) in the cohort.

We found 27 arm-level modifications (Figure 3), including gains in 3q, 5p and 8q, and losses in 3p and 8p chromosomal arms. Inside these 27 regions, we were able to identify 28 amplified and 48 deleted peaks (*q*-values < 0.25) that lodge 483 and 3270 loci, respectively. The largest peaks (affecting >100 genes) were Xq28 (amplification, 109), 1p13.2 (deletion, 152), 1p36.21 (deletion, 459), 2q36.2 (deletion, 281), 4p16.3 (deletion, 180), 11p15.5 (deletion, 188), 12q24.33 (deletion, 488), 14q11.2 (deletion, 268), 14q32.32 (deletion, 282) and 15q15.1 (deletion, 306). 

On the other hand, the narrowest peaks affecting a single locus were 7p11.2 (amplification; EGFR), 13q22.1 (amplification, LINC00393), 2q21.2 (deletion, RN7SKP154), 3p14.2 (deletion, PTPRG), 4q22.1 (deletion, RN7SKP248) and 10p11.21 (deletion, PARD3). 

Despite such a large number of compromised genes, according to our results, only 285 displayed positive selection signals, showing alterations in more than 10% of the patients. In terms of the proportions, the five most common somatic copy number (SCN)-altered genes in HNSCC were CDKN2A (deleted in 32.03% of patients), CDKN2B (deleted, 28.34%), PPFIA1 (amplified, 26.02%), FADD (amplified, 25.63%) and *ANO1* (amplified, 25.44%). With regards to known cancer drivers, 24 showed alterations, including the oral cell lineage transcription factors *TP63* (amplified, 21.94%) and *SOX2* (amplified, 20.97%), *CDKN2A*, *CCND1* (amplified, 24.27%), *PIK3CA* (amplified, 20.97%) and *EGFR* (amplified, 10.67%) (Figure 3).

We compared every tumour sample (diploid or somatic copy number modified) with the normal control samples to assess somatic copy number alterations (SCNAs) in terms of inducing alterations in the transcriptome. The results showed that 112 out of the 285 SCN-altered genes were differentially expressed in HNSCC samples. In total, 91 genes were upregulated while 21 showed lower levels of expression, 12 of them were cancer-related genes: two deleted (*CDKN2A* and *PTPRD*) and nine amplified (*EGFR* among others).

After obtaining DEGs and SCNA results, we have made a thorough calculation to relate expression and alterations in the number of copies. Table 7 shows those differently expressed genes in HNSCC, that have a copy number change in more than 10% of the cohort, with (log(FC) > 4 and < −4), and those that are classified in COSMIC.

### 3.4. Somatic Alteration Analysis

In the same HNSCC samples, around 94% of the VarScan-reported mutations had already been found in the MuTect2 results. The remaining 6% were distributed in different mutation categories, therefore demonstrating that VarScan2 can recall more mutations from every type due to its softer false positive filtering as: 276 frame shift deletions, 63 frame shift insertions, 147 in frame deletions, 5 in frame insertions, 2273 missense mutations, 193 nonsense mutations, 1 non-stop mutation, 71 splicing variants and 7 alterations in the transcription start site (TSS).

**Figure 4:**
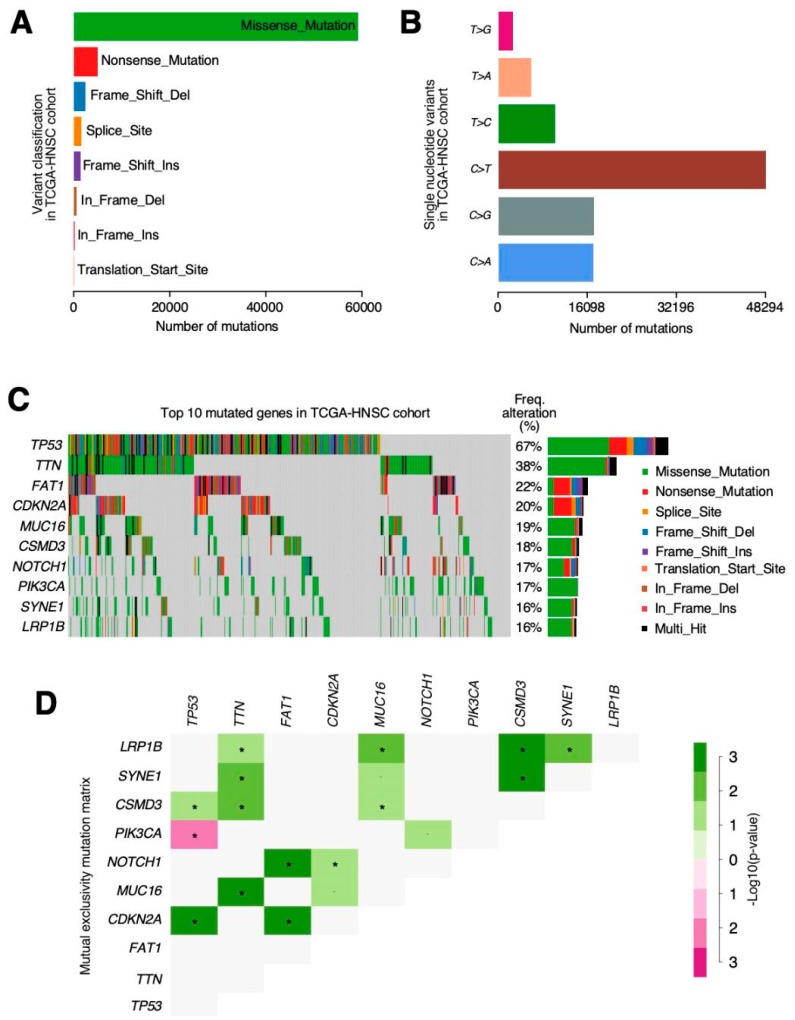
The MuTect2 and VarScan variant caller results are combined in order to facilitate the process of finding every possible alteration in the TCGA-HNSCC cohort. (**A**) Variants show the differences in abundancy across the cohort with missense being the largest represented group. (**B**) Nucleotide changes in single nucleotide variants (SNVs) show the differences in frequency, and the cases in which citosines are substituted by guanosines in the coding DNA strand are more likely to occur. (**C**) Oncoplot showing the top 10 most altered genes. The colours are associated with one class of variant and the percentage (%) of patients affected is shown on the graph. (**D**) Mutation mutual exclusivity matrix heatmap representation. The green colour is associated with a positive correlation whereas red indicates mutual exclusivity. *p*-value < 0.05 *.

The TCGA-HNSCC cohort was the ninth most mutated out of TCGA’s projects (Figure 4A). This indicated an average mutation rate per sample of 95 somatic alterations. In terms of proportion, missense variations were the most common, followed by nonsense and frameshift deletions, and cytosine SNPs (single nucleotide polymorphisms) (C > T, C > G or C > A) (Figure 4B) shows the highest frequencies. In fact, when looking at the top ten most mutated genes (Figure 4C) within the cohort, *FAT1* and *CDKN2A* are the only ones which were more affected by nonsense or frameshift deletions than missense ones. *TP53* was mutated in 72% of the tumour samples followed by *TTN* (39%), *FAT1* (23%) and *MUC16* (19%). 

Among these, we can highlight the mutual exclusivity pattern found between the *TP53* and *PIK3CA* mutations (Figure 4D); while, on the other hand, *CDKN2A* has shown co-occurrence with *TP53* and *FAT1* alterations. Finally, our results suggest different, very mutated protein domains in the cohort related to transcriptional control, signal transduction and, interestingly, G protein-coupled receptors (GPCR).

## 4. Discussion

A total of 528 patients were included in our study, 70.11% of patients were males, as had been the case in another similar study, however oral cavity tumours were more prevalent in our study, with 72.4% versus 62%. The tumour stage was predominantly (64.2%) advanced, (stage III and IV), similar to previous studies [15].

Transcriptional alterations analysis identified 3491 DEGs in HNSCC tumour samples when compared to healthy oral tissue. The oncogenes and tumour suppressor genes from the COSMIC cancer genes list were selected from the total DEGs, reducing these DEGs to 148. According to previous results, the more relevant DEGs were CDKN2A, BRCA2, CDK6, BRCA1, CDK4, TP63, EGFR, SOX2, KIT and ERBB4 [16,17]. 

The upregulated D-cyclin-dependent kinases, CDK4 and CDK6, were pro-tumorigenic proteins and core controllers of G1/S cell cycle phase transition [18]. Their tyrosin kinase domain phosphorylated the retinoblastoma protein (RB), impairing it from forming a complex with the E2F transcription factor. Once released, E2F induces the expression of the several genes that are necessary for the cell’s progression into the DNA synthesis phase. There are different regulators of CDK activity that could act as tumour suppressors, even by dually inhibiting cell cycle progression and enhancing pro-apoptotic factors. In this sense, HPV (+) cells show *P16^INK4A^* upregulation, a tumour suppressor gene transcribed from the *CDKN2A* locus [19]. This polypeptide inhibits CDK4/6 kinase activity and arrests the cell cycle at G1/S checkpoint. At the same time, P16^INK4A^ can also stabilize TP53, a potent tumour suppressor and pro-apoptotic protein, by sequestering the murine double murine 2 (MDM2) ubiquitin into the nucleus. The high levels of p16 in HPV+ tumours unlikely result in significant stabilisation of p53 or inhibition of the cell cycle, as RB and p53 are degraded because of viral genes E7 and E6 activity. On the other hand, HPV (−) cancers centre around genes that control cell motility, invasion, EMT and angiogenesis [20]. 

The upregulation of the epidermal growth factor receptor (EGFR) into tumour cells can intensify its signalling and promote both abnormal rates of cell proliferation and cell survival [21]. The EGFR gene belongs to the ERBB family of kinase receptor proteins alongside three other members: ERBB2, ERBB3 and ERBB4. Intriguingly, contrary to what was expected and published for other cancers [22,23], the ERBB4 gene is found deeply downregulated (log_2_(FC) = −3.67) in the HNSCC tumour samples. Other pathways, in which members are upregulated, are the PIK3CA/AKT/mTOR and WNT/B-catenin signalling axes, some of the Hippo pathway transductors and the DNA repair machinery members. It has been proven that these mechanisms were also implicated in previous HNSCC studies, not only by DEGs but also by other epigenetic biomarkers [24].

We also found recurrent repression of KIT receptor and other elements of its signalling route. This is not the first time this pro-tumorigenic pathway has been found to be downregulated in cancer. In thyroid carcinoma, lower levels of KIT positively correlate with more malignant phenotypes [25].

Functional analysis reveals that the more relevant signalling pathways in upregulated genes were those that were involved in tissue development, such as endoderm development, tissue morphogenesis, or skeletal system development. On the other hand, those downregulated genes were more implicated in the cell differentiation molecular pathway, salivary secretion, cornification or starch and sucrose metabolism. Altered molecular mechanism identification is useful for designing new treatment strategies such as immunotherapy, since these targeted therapies are effective against determined molecules [26].

In order to help to develop new therapies and programs for HNSCC prevention, as there is a lot of information in our analysis, we wanted to make our results more useful by performing a studies profile in HNSCC attending on localization. We could observe that, especially in oropharynx, DEGs’ number were significantly different (1209 genes exclusively different express in oropharynx) comparing results with the other localization, as none of the other group showed more than 224 DEGs. Although cautiously, these differences in DEGs results, the majority of the HPV (+) tumours are concentrated in the oropharynx group, where samples from the tonsil are included. Differential expression profiles developed by other authors, as well as by our group some years before, in HNSCC, revealed heterogeneity in results that reflects the nature of cancer [27,28].

Data obtained from survival analysis reveal different oncogenes overexpressed and whose higher expression correlates with worse survival. Other genes classified as tumour suppressors, whose lower expression correlates with worse survival but which, in the general cohort, are found with greater expression than normal samples. Concretely, EXT2 and IGF2BP2, are two known tumour suppressor genes whose highest expression correlates with worse survival. Taken altogether, there are genes classified as oncogenes or tumour suppressor genes in the COSMIC and that in our cohort show survival impacts that support this hypothesis, but there are some others that deviate from this theory, which behaviours show contrary to those expected genes that are classified as oncogenes, overexpressed and whose higher expression correlates with worse survival. CDKN2A, which is used in the diagnosis of HNSCC to check malignancy, has different behaviour in our results, as its overexpression is usually associated with better survival. It may be due to the fact that this correlation can be made if we are in context of HPV infection.

Analyses that report differential expression genes and somatic copy number alterations, show that is complicated to establish a cause-effect edge. Some genes are overexpressed and amplified, such as, for example, FOXL2, but there are other deleted genes that are over expressed, such as MUC4. This can be explained because, although there is a percentage of samples where the gene is not present, in the other, they have a tendency to increase its expression in comparison with normal tissue. The same can occur with amplified or repressed genes. Although a gene is amplified, there are other known mechanisms that act in a superior level that copy number alterations, like epigenetic changes or cotranscriptional repression.

Today’s primary challenge is to differentiate somatic mutations with oncogenic potential from all those which crop up later in a tumour’s lifetime without contributing to the tumour development. In this sense, the concept of positive selection has opened a new avenue for the discovery of novel tumorigenic roles in cancer. Mutations that improve the fitness of the tumour cells would be under positive selection pressure, meaning that they would recur at elevated rates across patients. This is the principle that is widely used to identify these signals and differentiate them from alterations with real oncogenic power [29].

SCNAs are very frequent genomic aberrations in cancer. Despite the fact that they directly affect the genome architecture, the main force through which they trigger their oncogenic potential is transcriptional deregulation. In fact, the amplifications of many oncogenes and the deletions of tumour suppressors were related to the cancer’s aggressiveness, endangering the patients’ survival.

In this study, we used the GISTIC2.0 algorithm results to identify recurrent SCNAs in the TCGA-HNSCC tumour samples. Our results demonstrated the co-amplification of segments 11q13 and 11q22, both embedding caspase cascade inhibitors, as has been previously described [8]. Modifications of 27 arm-level with gains in 3q, 5p and 8q, and losses in 3p and 8p chromosomal arms, have already been described in lung squamous cell carcinomas [30]. Taking into account the five most SCN-altered genes in HNSCC, deletion was observed in CDKN2A, CDKN2B, and amplification in PPFIA1, FADD and *ANO1.* These results concur with recent studies [31] and also with other known cancer driver amplifications, such as SOX2, PIK3CA, EGFR and CCDN1. SOX2 is a well-known oncogene that has already been shown SCNA in HNSCC and lung squamous cell carcinoma [32]. PIK3CA amplifications have been associated with an increased risk of local recurrence [33]. EGFR and CCDN1 amplification have been associated with clinical stage, tumour differentiation, and lymph node metastasis in HNSCC [31].

The TCGA mutation-calling pipeline offers four different methods to elucidate every single variation inside the tumour’s whole exome sequencing (WES) results. They are used in parallel and independently to better compare final results. In this sense, VarScan has been designed to be more permissive in the introduction of false positives, which was demonstrated to give a better performance [34] when dealing with “normal allele alteration” problems. On the other hand, MuTect2 is the algorithm with the most stringent false-positive filter [34]. These methods and others have been challenged in many reviews, showing that MuTect2 has the best performance dealing with both low- and high-frequency somatic mutation discovery [35,36]. In this study, we decided to use the combination of results from both the VarScan and MuTect2 pipelines.

The most relevant mutations in our cohort were *TP53* (72%) followed by *TTN* (39%), *FAT1* (23%) and *MUC16* (19%). TP53 has also been observed as the most prevalent mutation in previous studies [37,38] and also CDKN2A, EGFR and FAT1 were observed as being the most relevant [38]. Among all mutations, we highlighted the mutual exclusivity pattern found between the *TP53* and *PIK3CA* mutations. PIK3CA mutations have been associated with tobacco and alcohol consumption in previous studies [39]. Maybe, HPV status can explain this mutually exclusive mutations based on HPV status, as p53 mutations are very rare in HPV (+) cancers, while PIK3CA mutations are common, at least in a genetically well-defined subgroup of HPV (+) cancers [40].

Although these results do not concur with Babur et al. [41], the mutual exclusivity pattern is necessary in order to delineate the functional relations and involvement in common pathways of cancer-causing alterations. The identification of these patterns permits clinicians and scientists, not only to establish new tumour classifications or new preventive therapies, but also to design potential treatment targets and identify tumour vulnerabilities. Therefore, further analysis of mutual exclusivity patterns in HNSCC is still necessary [42].

cBioportal is a web application that simplifies data acquisition and processing from many high-quality publications, including the TCGA-HNSCC cohort. According to the cBioPortal analysis, the top five alteration genes were CDKN2A, CDKN2B, FGF3, FGF4, and FGF19 whereas in our results, those were CDKN2A, CDKN2B, PPFIA1, FADD, and ANO1. Theses discrepancies can be explained because there are two ways of using cBioportal: first, we can consult a dataset’s highlighting alterations in the “summary mode” or, second, a “by gene” query is available if we have a pre-selected genes list of our interest. cBioportal summary mode on TCGA-HNSCC “provisional” dataset show FGF3, FGF4 and FGF19 as copy number altered genes in 25% of the patients and no mention about PPFIA1, FADD or ANO1 genes. Nevertheless, the same platform when using the “by gene” query and the same dataset reports copy number alterations in these genes as 26% in the case of PPFIA1 and 25% in the cases of ANO1 and FADD.

We also have found differences in mutation data, while the top five genes in cBioPortal were TP53, FAT1, CDKN2A, NOTCH1, and PIK3CA, whereas in our results, they were TP53, TTN, FAT1, and MUC16. We did both summary and “by gene” queries in cBioportal and results were very similar as in copy number alteration matter. In this case, a reviewer search returned TP53, FAT1, CDKN2A, NOTCH1 and PIK3CA as the top mutated genes in the TCGA-HNSCC “provisional” dataset. There were no mentions about TTN or MUC16 mutation frequency, as we have reported. Once again, if we do a personalized query including all these genes, we face the same issue where TTN and MUC16 show larger mutation proportion (42% and 20%, respectively) than NOTCH1 or PIK3CA, both mutated in 18% of the samples. These discrepancies in cBioportal reports are intriguing and may be much better explained from its creator team and should not suppose any issue for the acceptance of our results as accurate and veracious.

Identification of those genes that are drivers in the tumorigenesis process is difficult as, even in the same tumour samples, different somatic alterations can be identified [43]. After analysing missense mutations and truncated mutations in oncogenes and tumour suppressors (results not shown), we have found that generally, in oncogenes, missense mutations are superior to truncated mutations but not in tumour suppressors, except from those more famous in HNSCC as CDKN2, FAT1 or PTEN. This means that missense mutations also can produce downregulation in a protein activity, so they are not always associated with gain of function. Some tumour suppressors analysed in this study were not described as tumour suppressors in HNSCC and it is possible that missense mutations that we have found were really random.

Although we were unable to assess the HPV infection with SCNA or somatic mutations, as only a small proportion were HPV (+), previous studies that have associated these alterations with HPV infections have shown enrichment mutations in HPV-negative in TP53, CDKN2A and PIK3CA, and also copy number gains in EGFR and CCND1, similar to our results [44]. Despite the already mentioned limited HPV (+) proportion, we performed differentially expression gene analysis in HPV (−) and HPV (+), obtaining similar results as other authors before us [45].

Clustering genes in similar signalling pathways or even targets similar to other cancers can help to determine the treatment with targeted drugs that are already available or under development. HNSCC classical classification is not always able to be associated with a determined prognostic. Genetic expression analysis, somatic mutations and SCNA can help us to determine patterns that will enable the tumoral behaviour to be determined or the possible targeted therapies to be identified.

## 5. Conclusions

Comprehensive analysis of HNSCC expression genes and somatic mutations allows us to identify deregulated genes and the most relevant molecular pathways altered in patients with HNSCC. The most prevalent mutations were found in TP53, TTN, FAT1 and MUC16. Other relevant results were the mutual exclusivity patterns between the TP53 and PIK3CA mutations and CDKN2A co-occurrence with TP53 and FAT1. Functional studies confirmed that deregulated genes play a role in mechanisms involved in cell differentiation and tissue development. Together, these new findings help us to develop new therapies and programs for the prevention of HNSCC.

## Figures and Tables

**Table 1:** 20 most relevant differentially expressed genes (DEGs) exclusively in oral cavity tumour samples compared with normal tissue.

Gene	LogFC	*p*-Value
*FADS2*	−18,712,923	4.87 × 10^−29^
*IGJ*	−16,587,232	1.47 × 10^−22^
*ADAMTSL3*	−16,583,309	2.57 × 10^−22^
*IL17RD*	−14,657,516	1.75 × 10^−17^
*IL34*	−13,584,484	5.26 × 10^−15^
*SMOC1*	175,035,565	1.07 × 10^−14^
*ALDH6A1*	−13,347,877	1.67 × 10^−14^
*RARB*	−13,085,358	6.18 × 10^−14^
*MPV17L*	−13,145,417	7.70 × 10^−14^
*TNFRSF19*	−12,952,743	1.06 × 10^−13^
*TMC4*	−12,528,259	7.59 × 10^−13^
*PRELP*	−12,484,188	9.27 × 10^−13^
*C1orf115*	−12,205,336	3.31 × 10^−12^
*SELE*	−1,218,887	3.51 × 10^−12^
*ADCY5*	−12,226,496	4.06 × 10^−12^
*FAM184B*	−12,319	4.48 × 10^−12^
*SIX1*	−12,128,568	4.66 × 10^−12^
*PLS1*	−12,117,518	4.85 × 10^−12^
*TSHZ2*	−12,054,309	6.57 × 10^−12^
*MYL4*	152,505,375	1.05 × 10^−11^

**Table 2:** 20 most relevant DEGs exclusively in oropharynx tumour samples compared with normal tissue.

Gene	LogFC	*p*-Value
*KRT81*	−33,881,326	7.40 × 10^−43^
*TDRD10*	372,051,812	1.22 × 10^−42^
*RORB*	355,120,316	2.02 × 10^−40^
*LCE2A*	−33,411,619	1.71 × 10^−38^
*YBX2*	321,467,402	1.30 × 10^−34^
*GPAT2*	310,447,612	1.07 × 10^−32^
*BCAN*	309,645,231	1.68 × 10^−32^
*CDKN2C*	30,654,531	3.96 × 10^−32^
*MGAT3*	286,859,034	7.55 × 10^−29^
*HRNR*	−26,945,379	7.58 × 10^−29^
*CLGN*	281,706,716	5.90 × 10^−28^
*IL17RB*	274,622,975	8.29 × 10^−27^
*EPHA7*	261,626,827	8.18 × 10^−25^
*RIBC2*	259,473,711	1.98 × 10^−24^
*FMO3*	258,820,947	2.17 × 10^−24^
*HPN*	−23,997,095	3.88 × 10^−23^
*C13orf33*	−23,590,997	3.91 × 10^−23^
*C19orf57*	250,444,482	4.37 × 10^−23^
*SOX8*	240,992,451	1.26 × 10^−21^
*STEAP4*	−22,640,948	1.43 × 10^−21^

**Table 3:** 20 most relevant DEGs exclusively in hypopharynx tumour samples compared with normal tissue.

Gene	logFC	*p*-Value
*DEFA6*	868,622,546	1.65 × 10^−121^
*KCNH1*	244,008,193	1.00 × 10^−24^
*PCYT1B*	238,001,899	7.97 × 10^−24^
*IGSF11*	214,408,262	3.48 × 10^−20^
*DEFB4A*	−16,847,413	1.01 × 10^−19^
*DLGAP1*	210,854,558	1.23 × 10^−19^
*PCDH19*	207,400,768	2.97 × 10^−19^
*SLC4A8*	179,379,726	3.00 × 10^−15^
*RGS6*	177,738,587	8.93 × 10^−15^
*FREM2*	173,946,254	1.45 × 10^−14^
*TMEM27*	172,587,192	3.47 × 10^−14^
*ALPK2*	−14,096,238	5.27 × 10^−14^
*DAZ1*	170,462,856	5.27 × 10^−14^
*BDNF*	16,819,588	9.36 × 10^−14^
*CES4*	164,630,763	2.77 × 10^−13^
*CHODL*	163,016,037	4.34 × 10^−13^
*GFRA3*	161,613,625	7.17 × 10^−13^
*CES1*	159,875,222	9.05 × 10^−13^
*TET1*	159,391,324	1.28 × 10^−12^
*GPX2*	156,667,856	2.28 × 10^−12^

**Table 4:** 20 most relevant DEGs exclusively in lip tumour samples compared with normal tissue.

Gene	logFC	*p*-Value
*MEG3*	−20,994,236	4.39 × 10^−23^
*ADARB2*	−19,759,467	4.32 × 10^−20^
*ISL1*	−17,718,926	1.14 × 10^−16^
*GDF15*	−1,733,074	2.39 × 10^−16^
*LRRC55*	171,507,607	8.43 × 10^−13^
*C11orf70*	−14,775,272	4.09 × 10^−12^
*PKDCC*	−14,416,166	1.03 × 10^−11^
*HNF4G*	−14,089,033	5.52 × 10^−11^
*DOCK3*	−13,914,239	6.44 × 10^−11^
*WSCD1*	−13,655,624	1.54 × 10^−10^
*COL4A3*	−13,635,425	1.86 × 10^−10^
*NPHP1*	−13,532,494	1.99 × 10^−10^
*LRRC23*	−1,329,676	3.54 × 10^−10^
*RNF183*	−13,386,114	4.65 × 10^−10^
*CCDC146*	−13,154,093	5.58 × 10^−10^
*PTP4A3*	−13,117,555	5.83 × 10^−10^
*DNAH2*	−13,151,428	5.99 × 10^−10^
*CXCL5*	14,316,611	1.22 × 10^−09^
*CPAMD8*	−12,854,897	1.52 × 10^−09^
*MRGPRX3*	142,602,231	1.54 × 10^−09^

**Table 5:** Survival analysis of DEGs in HNSCC. K-M *p* va. > 0 means high expression with poorer prognosis and <0 means lower expression with poorer prognosis. ONC: oncogene, TSG: tumour suppressor gene.

Gene	K-M P.VAL	Cosmic Condition	*p*-Value	FDR
*ABL2*	0.02114593	ONC	3.41 × 10^−10^	1.97 × 10^−09^
*CCR7*	−0.0004892	ONC	1.21 × 10^−06^	4.90 × 10^−06^
*CD79A*	−0.0034022	ONC	1.76 × 10^−16^	1.54 × 10^−15^
*CDK4*	0.00832232	ONC	1.35 × 10^−07^	6.11 × 10^−07^
*CDK6*	0.01344407	ONC	6.01 × 10^−12^	3.96 × 10^−11^
*EGFR*	0.01370546	ONC	1.43 × 10^−09^	7.86 × 10^−09^
*ETV4*	0.00467991	ONC	1.33 × 10^−08^	6.68 × 10^−08^
*E × T2*	0.00523221	TSG	3.26 × 10^−07^	1.41 × 10^−06^
*FGFR4*	0.02339692	ONC	5.87 × 10^−17^	5.34 × 10^−16^
*FSTL3*	0.04179603	ONC	6.87 × 10^−26^	9.67 × 10^−25^
*HIF1A*	0.00962668	ONC	4.44 × 10^−07^	1.89 × 10^−06^
*HLF*	−0.0229118	ONC	1.18 × 10^−24^	1.57 × 10^−23^
*HMGA2*	0.00936317	ONC	7.48 × 10^−67^	2.54 × 10^−65^
*HOXC13*	0.00695101	ONC	1.21 × 10^−17^	1.15 × 10^−16^
*IGF2BP2*	0.00020864	TSG	3.69 × 10^−29^	5.73 × 10^−28^
*JAK3*	−0.0117944	ONC	4.94 × 10^−09^	2.57 × 10^−08^
*LCK*	−0.0272594	ONC	4.74 × 10^−07^	2.01 × 10^−06^
*PDGFB*	0.0032238	ONC	3.99 × 10^−07^	1.71 × 10^−06^
*POU2AF1*	−0.0263913	ONC	4.14 × 10^−11^	2.57 × 10^−10^
*RET*	0.04810527	ONC	1.01 × 10^−08^	5.10 × 10^−08^
*RHOH*	−0.0336722	TSG	7.65 × 10^−07^	3.17 × 10^−06^
*ROS1*	0.032848	ONC	2.20 × 10^−35^	4.02 × 10^−34^
*SIX1*	0.01805839	ONC	7.85 × 10^−11^	4.76 × 10^−10^
*SOCS1*	−0.0433087	TSG	5.12 × 10^−21^	5.82 × 10^−20^
*TCL1A*	−0.0164834	ONC	1.68 × 10^−14^	1.32 × 10^−13^

**Table 6:** DEGs in HPV (+) and HPV (−) tumour samples.

Gene	logFC	*p*-Value
*ZFR2*	743,557,827	0
*KRT38*	685,090,585	0
*SMC1B*	646,035,753	0
*TAF7L*	632,737,642	0
*STAG3*	586,115,531	3.19 × 10^−305^
*INSM1*	571,577,721	2.08 × 10^−287^
*ABCA17P*	574,001,163	6.07 × 10^−287^
*ZNF541*	540,074,496	2.27 × 10^−257^
*NEFH*	518,927,943	2.98 × 10^−239^
*KEL*	51,354,914	1.50 × 10^−232^
*TDRD9*	507,100,644	1.63 × 10^−226^
*SYCP2*	496,943,561	4.06 × 10^−219^
*TCAM1P*	483,751,729	5.47 × 10^−205^
*RORB*	477,878,379	4.33 × 10^−200^
*GRIN2A*	475,032,624	5.33 × 10^−199^
*UPB1*	477,610,842	1.63 × 10^−196^
*TDRD10*	476,235,561	5.24 × 10^−196^
*SOX30*	455,517,399	5.03 × 10^−180^
*PAX1*	446,391,442	2.64 × 10^−175^
*C11orf92*	442,147,634	7.60 × 10^−172^

**Table 7:** COSMIC DEGs HNSCC also with a copy number changed in more than 10% of the cohort. DEL: deleted, AMP: amplified.

Gene	logFC	*p*-Value	FDR	Patients%	Condition
*ADIPOQ*	−5.46	2.00 × 10^−194^	2.05 × 10^−192^	198,058,252	AMP
*CDKN2A*	4.15	1.08 × 10^−25^	8.69 × 10^−25^	32,038,835	DEL
*COL22A1*	4.36	1.64 × 10^−27^	1.44 × 10^−26^	100,970,874	AMP
*FGF19*	9.34	7.97 × 10^−68^	2.40 × 10^−66^	244,660,194	AMP
*FGF3*	7.42	6.14 × 10^−52^	1.19 × 10^−50^	250,485,437	AMP
*FOXL2*	4.56	8.80 × 10^−29^	8.41 × 10^−28^	100,970,874	AMP

## Abbreviations

DEG	Differentially expressed genes
FDR	False discovery range
GDC	Genomic Data Commons
HNSCC	Head and neck squamous cell carcinoma
HPV	Human papilloma virus
RNA-seq	RNA sequencing
MAF	Mutations annotation format
HR-HPV	High-risk human papilloma virus
SCNA	Somatic copy number alterations
SNP	Single nucleotide polymorphisms
TCGA	The cancer genome atlas
TSS	Transcription start site
